# Metabolic Adaptation during nab-Paclitaxel Resistance in Pancreatic Cancer Cell Lines

**DOI:** 10.3390/cells9051251

**Published:** 2020-05-19

**Authors:** Lukas M. Braun, Simon Lagies, Jessica Guenzle, Stefan Fichtner-Feigl, Uwe A. Wittel, Bernd Kammerer

**Affiliations:** 1 Center for Biological Systems Analysis ZBSA, Albert-Ludwigs-University Freiburg, 79104 Freiburg, Germany; lukas.braun@uniklinik-freiburg.de (L.M.B.); simon.lagies@zbsa.uni-freiburg.de (S.L.); 2 Department of General- and Visceral Surgery, University of Freiburg Medical Center Faculty of Medicine, 79106 Freiburg, Germany; jessica.guenzle@uniklinik-freiburg.de (J.G.); stefan.fichtner@uniklinik-freiburg.de (S.F.-F.); 3 Spemann Graduate School of Biology and Medicine, Albert-Ludwigs-University Freiburg, 79104 Freiburg, Germany; 4 Institute of Biology II, Albert-Ludwigs-University Freiburg, 79104 Freiburg, Germany; 5 BIOSS Centre for Biological Signalling Studies, University of Freiburg, 79104 Freiburg, Germany

**Keywords:** pancreatic ductal adenocarcinoma, pancreatic cancer, PDAC, metabolomics, metabolic reprogramming, chemotherapy resistance, nab-Paclitaxel, GC/MS

## Abstract

Pancreatic ductal adenocarcinoma (PDAC) correlates with high mortality and is about to become one of the major reasons for cancer-related mortality in the next decades. One reason for that high mortality is the limited availability of effective chemotherapy as well as the intrinsic or acquired resistance against it. Here, we report the impact of nab-paclitaxel on the cellular metabolome of PDAC cell lines. After establishment of nab-paclitaxel resistant cell lines, comparison of parental and resistant PDAC cell lines by metabolomics and biochemical assessments revealed altered metabolism, enhanced viability and reduced apoptosis. The results unveiled that acute nab-paclitaxel treatment affected primary metabolism to a minor extent. However, acquisition of resistance led to altered metabolites in both cell lines tested. Specifically, aspartic acid and carbamoyl-aspartic acid were differentially abundant, which might indicate an increased de novo pyrimidine synthesis. This pathway has already shown a similar behavior in other cancerous entities and thus might serve in the future as vulnerable target fighting resistance acquisition occurring in common malignancies.

## 1. Introduction

Pancreatic ductal adenocarcinoma (PDAC) is a highly aggressive and fast growing type of cancer [1], which is associated with a poor prognosis [2] and is predicted to become the second-most cause of cancer-associated mortality by 2030 [3]. The five-year survival rate of PDAC patients changed marginally during the last years from below five percent to ten percent [4,5]. PDAC has high frequencies of oncogenic mutations driving cancer survival, growth, resistance to therapy, and supporting anabolic metabolic pathways [6]. The very low survival rates associated with PDAC are mainly due to the very rapid progression of pancreatic cancer [7], the extremely rare exhibition of any specific symptoms [6] and the lack of any powerful biomarkers for early disease detection [8]. The hunt for reliable biomarkers is highly difficult and today, the only available clinical tumor marker is carbohydrate antigen 19-9 (CA19-9), which clearly indicates an urgent need of new biomarkers to reliably detect PDAC at an early disease state to improve patients’ prognoses [9,10]. The detection of mutations in cell-free DNA [11], tumor-specific antibodies [12], secreted exosomes [8], or metabolites [13], like free modified nucleosides, are all promising candidates for future biomarkers. When metabolites could be used as biomarkers in PDAC, one can expect that the metabolome is also disturbed. Indeed, metabolism is reprogrammed in PDAC [14]. The most prominent metabolic switch is known as the “Warburg Effect” [15], in which lactate production is favored over oxidative phosphorylation. Cancer cells also depend on reprogramming glucose and glutamine import to maintain carbon pools for building macromolecules [14]. 

There are different therapies for PDAC treatment available, but effects are limited [3]. Three of the well-studied or new drugs are 5-fluorouracil (5-FU), gemcitabine and nab-paclitaxel (nanoparticle albumin-bound paclitaxel). The pyrimidine analog 5-FU [16] is the oldest of these compounds but did only show limited effects as a monotherapy [17]. Combinations with other compounds exhibited a promising role in PDAC treatment [18,19]. Gemcitabine, a pyrimidine antimetabolite [20], was presented as a single-agent compound in pancreatic cancer [21] and showed better clinical responses and median overall survival (OS) [22] compared to 5-FU. However, the survival benefit of gemcitabine treatment was limited [23]. Recently, the addition of nab-paclitaxel to gemcitabine improved the survival in PDAC patients when compared to gemcitabine alone [24,25]. 

Unlike inhibition of DNA synthesis and DNA damage induction by 5-FU [26] and gemcitabine [27], nab-paclitaxel leads to cell cycle arrest through microtubule stabilization and programmed cell death [28]. Despite multiple treatment options, PDAC is one of the most resistant cancer types to chemotherapy explaining the frequent treatment failure and non-responding tumors [29,30]. Additionally, most of the treatment failures are due to primary or acquired resistances [6] in uptake transporters [31,32,33], metabolizing enzymes [31], repair enzymes for DNA damage [34], or apoptosis pathways [35,36].

We investigated metabolic alterations in PDAC cell lines upon exposure to the chemotherapeutic compound nab-paclitaxel. Moreover, we analyzed the metabolic adaptations of nab-paclitaxel-resistant PDAC cell lines, as shown for gemcitabine resistance in previous publications [37,38]. Our aim was further to identify metabolic vulnerabilities in resistant PDAC cells compared to non-resistant PDAC cells. These could serve as novel future targets to overcome treatment resistances in pancreatic cancer. 

## 2. Materials and Methods 

### 2.1. Cell Culture 

MiaPaCa-2 (CRL-1420™) and Panc-1 (CRL-1469™) were purchased from the American Type Culture Collection (ATCC, Manassas, VA, USA). Resistant cell lines were established as described below. All cell lines were cultured in DMEM high-glucose medium (Gibco, Carlsbad, CA, USA) supplemented with 10% fetal bovine serum (Gibco). Culture medium for Panc-1/NPR cells was supplemented with 10 nM nab-paclitaxel (Celgene, Summit, NJ, USA) and culture medium for MiaPaCa-2/NPR cells was supplemented with 5 nM nab-paclitaxel. All cell lines were grown at 37 °C and 5% CO_2_ in a humidified atmosphere. Characteristics of parental cell lines are summarized in Lagies et al. [13].

### 2.2. Viability Assay and IC50 Calculation

Three thousand cells/well were seeded in a total volume of 100 µL culture medium in 96-well plates and incubated o/n at the respective culture conditions. The next day, cells were treated as indicated and incubated for 72 h before addition of 10 µL resazurin (R&D Systems, Minneapolis, MN, USA) for 4 h. Fluorescence was read at 544 nm excitation and 590 nm emission. Cell viability was calculated as relative viability with reference to untreated control (100%). The IC50 values were calculated using GraphPad Prism software V5 (GraphPad Software Inc., San Diego, CA, USA).

### 2.3. Establishing Chemotherapy-Resistant Pancreatic Cancer Cell Lines

Chemotherapy resistant cell lines were established from MiaPaCa-2 and Panc-1 cells. 100,000 cells were seeded in 1 mL in 12-well plates and incubated o/n before treatment. Cells were shock-treated with high-dose chemotherapy (100 × IC50-concentration) for 24 h. The next day, cells were treated with 0.5 × IC50 chemotherapy concentrations. Culture medium and treatment were replaced twice a week and gradually increased when cells were growing well. The cells were transferred into 75-cm^2^ tissue culture flasks for further culturing, split when approximately 75% confluent and directly seeded in medium supplemented with chemotherapy. All resistant cell lines were cultured in culture medium with chemotherapy for at least 100 days before IC50 determination.

### 2.4. Cell Death Assay

For the cell death assay, 2.5 × 10^5^ cells were seeded in 6-well plates in 2 mL culture medium o/n and treated with chemotherapy for 72 h. Cells were harvested, washed with 1 × PBS and incubated with 50 µL annexin-V Ax647 (Applied Biosystems, Waltham, MA, USA) (1:20 in binding buffer (10 mM HEPES pH7.4, 140 mM NaCl, 2.5 mM CaCl_2_, 0.1% BSA, H_2_O to 100 mL)) on ice for 15 min. 450 µL of 20 µg/mL propidium iodide (Sigma, St. Louis, MO, USA) was added to each sample. The samples were acquired using a BD LSR Fortessa (BD Biosciences, Franklin lakes, NJ, USA) and analyzed using the FlowJo™V10 software.

### 2.5. Untargeted GC/MS-Based Metabolic Profiling

For the untargeted GC/MS-based metabolic profiling, 1 × 10^6^ cells were seeded in 10-cm culture dishes in 8 mL culture medium and treated with chemotherapy o/n for 72 h before metabolomics analysis. Metabolic profiling was conducted as reported previously [39]. In short, each plate was washed twice with 5 mL cold 0.9% NaCl. Metabolism was quenched by adding 1.5 mL ice-cold extraction buffer (MeOH/H_2_O 9:1 (*v*/*v*)) containing internal standards; then, cells were scraped off and snap-frozen in liquid nitrogen. After mechanical lysis, metabolite pellets were derivatized by silylation and methoxyamination. Samples were analyzed in randomized order by GC-EI-MS (Agilent 7890A/5975C). Conserved mass spectral features [40] were matched to different libraries in regard to retention index and mass spectrum. After normalization to internal standard and peak-sum, statistical analyses were performed by MetaboAnalyst 4.0 [41]. For principal component analysis and heat map generation, values were range scaled. ANOVA results were corrected by FDR multiple testing correction followed by Tukey’s HSD post-hoc test.

### 2.6. Statistical Analysis

For other than the untargeted metabolomics profiling data, statistics were performed using the GraphPad prism software V5. Normally distributed data were compared using an unpaired *t*-test. Data are displayed as mean ± standard error of the mean. A *p*-value < 0.05 was considered as statistically significant. Significances were shown with symbols (* *p* < 0.05, ** *p* < 0.01, *** *p* < 0.001). No repeated measurements from the same sample were performed with the exception of QC samples in GC/MS analyses.

### 2.7. Data Availability

Results of GC/MS analyses are provided in Supplementary Data files SD1 and SD2. 

## 3. Results and Discussion

### 3.1. Treatment of Pancreatic Cancer Cells Lines with nab-Paclitaxel Resulted in Few Metabolic Alterations

**Figure 1:**
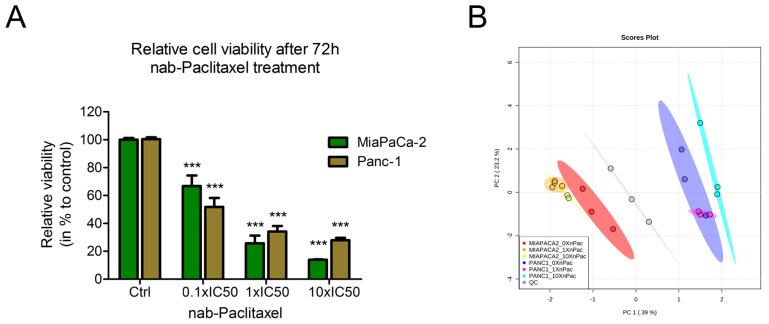
(**A**) Relative viability of nab-paclitaxel treated cells with 0.1 × IC50, 1 × IC50 and 10 × IC50 concentrations for 72 h. Control (Ctrl) treatment describes vehicle application. The viability of cells was calculated in percent relative to control treatment. Bar charts display mean ± standard error of the mean (*n* = 9). A *p*-value of *p* < 0.05 was considered as statistically significant (*** indicates *p* < 0.001). (**B**) Principal component analysis of endometabolome GC/MS profiling of PDAC cell lines upon treatment with nab-paclitaxel. 0 × nPac: untreated control, 1 × nPac: IC50 concentration, 10 × nPac: ten-fold IC50 concentration. Quality control samples, consisting of equal volumes of all samples, were included into the analysis. Analysis was performed after 72 h treatment. *n* = 3.

To investigate the metabolic effects of chemotherapy treatment in pancreatic cancer cells lines, the IC50 concentrations of nab-paclitaxel were determined in the PDAC cell lines MiaPaCa-2 and Panc-1 (4.1 pM and 7.3 pM). The cells were treated with increasing concentrations of chemotherapy (0.1 × IC50, 1 × IC50 and 10 × IC50 concentration) and cell viability was measured 72 h after treatment. The viability of both cell lines significantly decreased in a dose-dependent manner compared to the control treatment (Figure 1A). The concentrations analyzed for viability were the same as applied to the cells in metabolomics experiments. 

Following, chemotherapy treated cells were subjected to untargeted GC/MS-based metabolic profiling. Applying two-dimensional principal component analysis (PCA), revealed global changes between the cell lines (Figure 1B). Despite these general differences between the cell lines, only the ten-fold IC50 concentration led to a discrimination from the corresponding control (Figure 1B).

**Figure 2:**
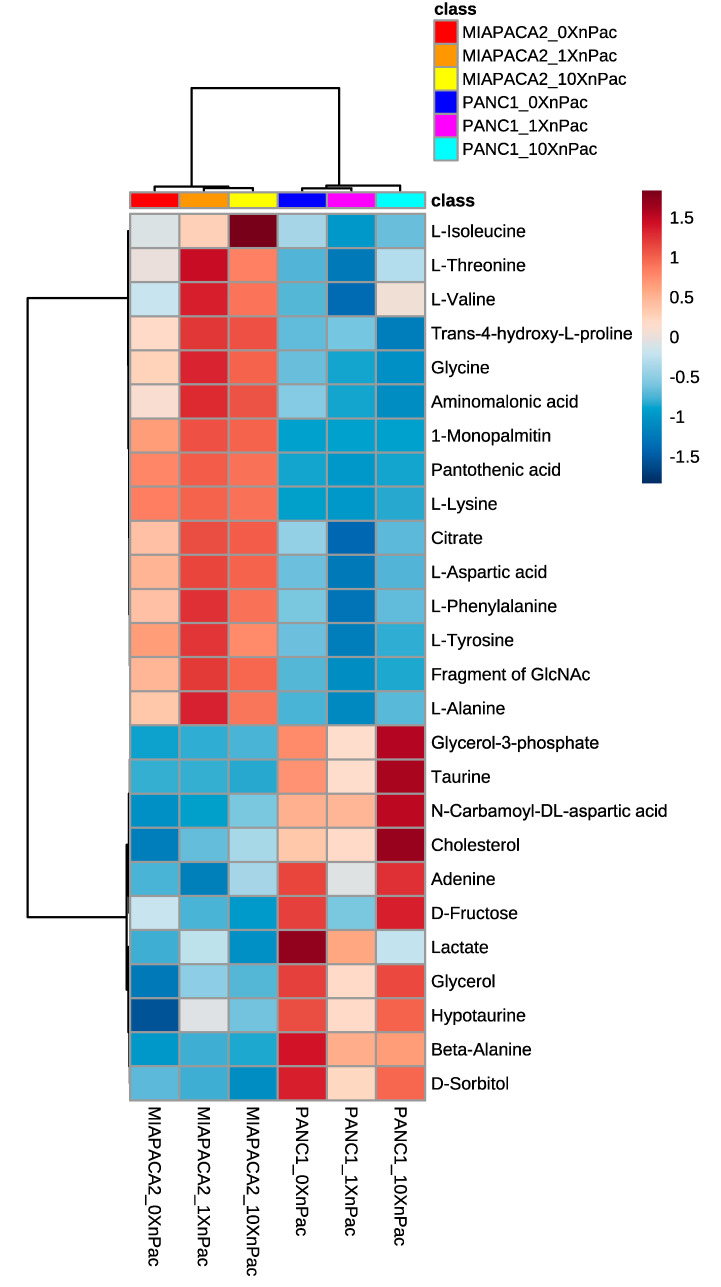
Heat map of metabolic, GC/MS-based profiling of PDAC cell lines upon treatment with chemotherapy. Significantly altered metabolites in MiaPaCa-2 and Panc-1 cell lines upon nab-paclitaxel treatment for 72 h. 0 × nPac: untreated control, 1 × nPac: IC50 concentration, 10 × nPac: ten-fold IC50 concentration. Range-scaled z-scores are shown. *n* = 3.

Figure 2 shows a heat map with z-scores of all intracellular altered metabolites in MiaPaCa-2 and Panc-1 cells after nab-paclitaxel treatment. The clustering in this heat map indicates that major changes were caused by differences between both cell lines and were not due to nab-paclitaxel treatment. This result confirms the observation obtained by PCA. Specifically, several amino acids were higher in MiaPaCa-2 cells, which might account for their higher proliferation rate in vitro [42,43], which is also maintained when transplanted into mice [44]. On the contrary, sorbitol and fructose, metabolites of the polyol pathway [45], are in general higher in the Panc-1 cell line. High expression of both enzymes involved in polyol metabolism has been correlated with a mesenchymal phenotype [46], and Panc-1 cells show a high abundance of vimentin and low levels of E-cadherin, suggesting such a mesenchymal phenotype [47].

Nab-paclitaxel treatment did only have slight effects on cellular metabolism. Intracellular levels of polyol-pathway intermediates revealed its inactivation upon nab-paclitaxel application. Additionally, an increase of taurine upon chemotherapy application confirms this hypothesis [48]. Within all analyzed amino acids, only isoleucine significantly increased intracellularly after nab-paclitaxel treatment, potentially due to decreased protein synthesis [49].

Application of the anti-metabolite gemcitabine to MiaPaCa2 and Panc-1 cells resulted in substantial metabolic changes, as shown by starker differentiation in the PCA (Figure S1A) and different clustering of altered metabolites (Figure S1B). This indicates that nab-paclitaxel does not have such a strong influence on the metabolome as other chemotherapeutic agents do.

### 3.2. Chemotherapy-Resistant PDAC Cell Lines Had Higher IC50-Values Compared to Their Parental Cell Lines

**Figure 3:**
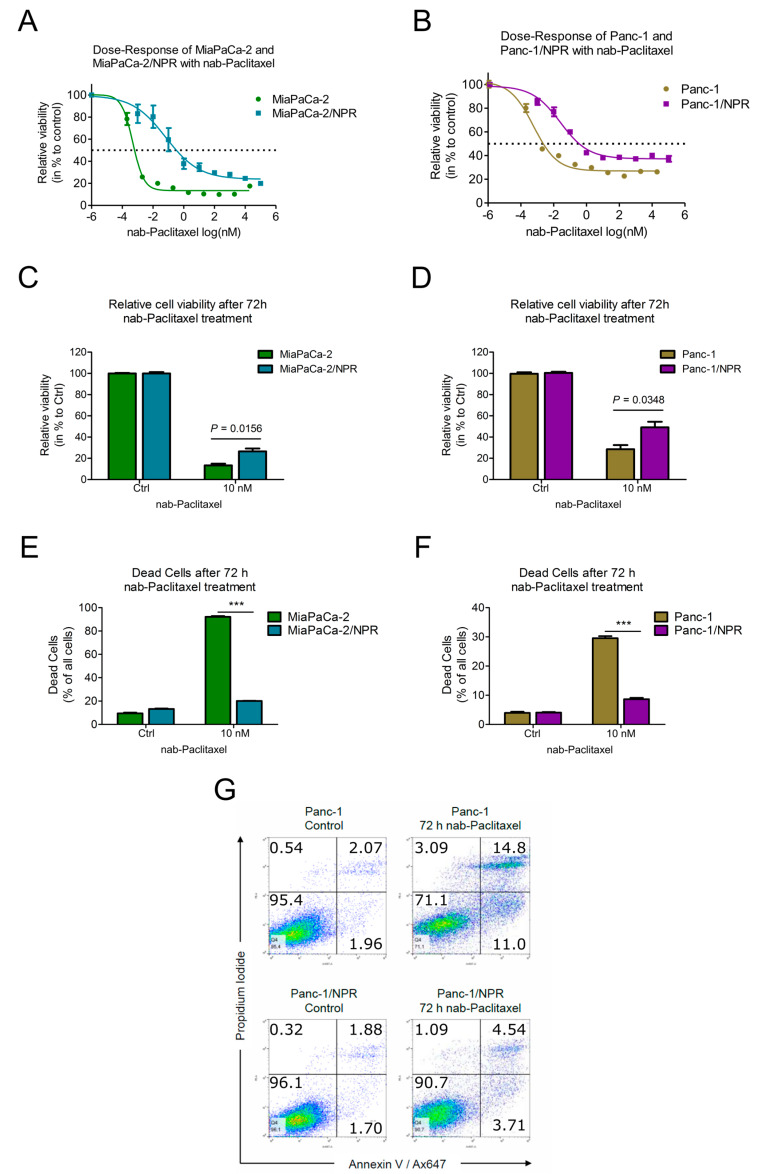
Viability and apoptosis of chemotherapy-resistant and parental cell lines after treatment. (**A**) Dose–response curves for MiaPaCa-2/NPR and MiaPaCa-2 cells upon nab-paclitaxel treatment. (**B**) Dose–response curves of Panc-1/NPR and Panc-1 cells upon nab-paclitaxel treatment. Dose–response was calculated relative to control after 72 h treatment. Dotted line indicates 50% viability. Results are displayed as mean ± standard error of the mean (*n* = 9). (**C**,**D**) relative viability of parental and resistant MiaPaCa-2 and Panc-1 cells upon nab-paclitaxel treatment (10 nM). (**E**,**F**) Bar charts showing dead cells (Annexin V^+^) as percent of all analyzed cells for different cell lines after control or chemotherapy treatment (10 nM). Bar charts display mean ± standard error of the mean (*n* = 3). Data were compared by unpaired *t*-test. A *p*-value of *p* < 0.05 was considered as statistically significant (*** indicates *p* < 0.001). (**G**) Representative FACS plots from F of resistant and parental Panc-1 cells upon control or nab-paclitaxel treatment. Cells were stained with anti-Annexin V (x-axis) and PI (y-axis).

The nab-paclitaxel-resistant cell lines MiaPaCa-2/NPR and Panc-1/NPR were selected for at least 100 days, as described in the methods section. The resistant cells and their corresponding parental cell lines were treated with increasing doses of chemotherapy for 72 h before viability analysis. In all cell lines, the viability decreased in a dose-dependent manner, whereas the viability of the resistant cell lines was always higher than the viability of the parental cell line (Figure 3A,B). The IC50-values were calculated and are summarized in Table 1. Fold change analysis of the IC50 of the resistant cells vs. the IC50 of the parental line indicated elevated IC50-values in all resistant cell lines. Application of 10 nM nab-paclitaxel resulted in significant increases of relative viability in both resistant cell lines (Figure 3C,D).

### 3.3. Resistant Cells Showed Less Apoptosis upon Chemotherapy Treatment 

In order to further confirm the acquired resistance, both parental and resistant cell lines were treated with 10 nM nab-paclitaxel for 72 h and analyzed for apoptosis by fluorescence activated cell sorting (FACS). Annexin V^+^ and Annexin V^+^/PI^+^ cells were summarized as dead cells and are given in percent of all analyzed cells after removal of doublets. Ten nanomolar (10 nM) nab-paclitaxel was used as the viability assay measures the redox state and disturbances in this may be insufficient to induce apoptosis. In MiaPaCa-2 cells treated with nab-paclitaxel, 90% of all cells underwent apoptosis, with only 20% dead cells in the MiaPaCa-2/NPR cell line (Figure 3E). Treatment with nab-paclitaxel resulted in 30% dead Panc-1 cells, whereas only 10% of all Panc-1/NPR cells underwent apoptosis (Figure 3F). Taken together, both chemotherapy-resistant cells showed significantly fewer dead cells after chemotherapy treatment compared to the parental cell lines. Representative FACS plots for parental and resistant PDAC cell line Panc-1 with or without nab-paclitaxel treatment are depicted in Figure 3G.

### 3.4. Metabolic Analysis of Chemotherapy-Resistant PDAC Cell Lines

**Figure 4:**
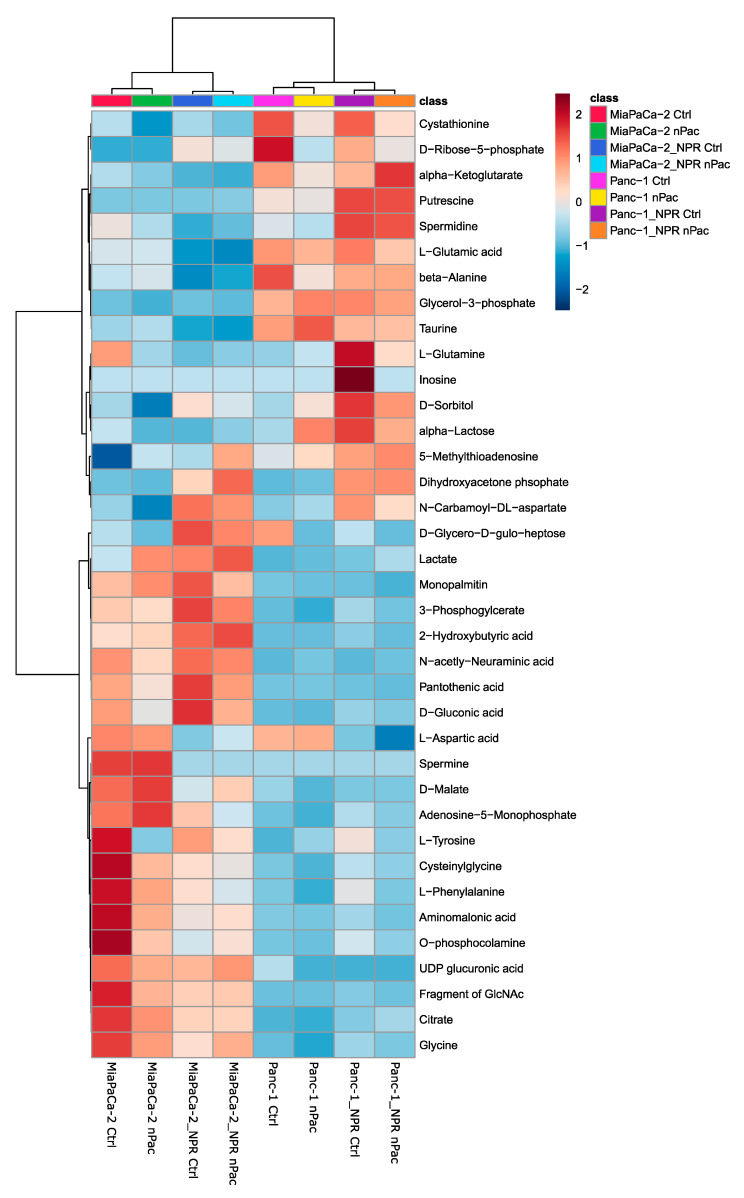
Heat map of significantly altered metabolites in chemotherapy-resistant and parental cell lines after chemotherapy treatment. The heat map describes all significantly altered metabolites identified by untargeted GC/MS profiling in chemotherapy-resistant and parental PDAC cell lines upon application of nab-paclitaxel. Cell lines were treated for 72 h. nPac: nab-Paclitaxel treated; Ctrl: vehicle treated. Range-scaled z-scores are shown. *n* = 3.

The cell lines MiaPaCa-2/NPR and Panc-1/NPR, as well as MiaPaCa-2 and Panc-1, were treated with control or nab-paclitaxel for 72 h. Afterwards, primary metabolites were assessed as mentioned previously. PCA revealed again that cell line differences were more prominent than the impact of nab-paclitaxel (Figure S2). The heat map depicts all significantly altered metabolites, visualized as range-scaled z-scores, found by metabolome GC/MS analysis (Figure 4). The clustering indicates that the strongest difference was between Panc-1 cell lines and MiaPaCa-2 cell lines, regardless if resistant or not. The second branch in the cluster analysis discriminated parental cell lines from resistant cell lines, while the least effects were observed by acute nab-paclitaxel treatment (Figure 4).

Elevation of lactate as well as the decrease of citrate and malate in the MiaPaCa-2/NPR cell line compared to its parental cell line suggests a switch to aerobic glycolysis. Putrescine and spermidine levels showed that Panc-1 cells had an activated polyamine pathway, however, the levels are even higher in the nab-paclitaxel resistant cell line Panc-1/NPR [50]. Increased sorbitol suggests the polyol pathway being activated in Panc-1/NPR cells and in MiaPaCa-2/NPR cells [46]. The decreased levels of pantothenic acid in Panc-1 cells might indicate a reduced demand to build biomolecules [51], possibly due to a lower proliferation rate [42,43]. Accordingly, MiaPaCa-2 cell lines had in general higher intracellular levels of amino acids, which could be because of a greater need for biomolecules due to faster proliferation compared to Panc-1 cell lines [52]. In line with this is the generally higher abundance of “fragment of GlcNAc”, an indistinguishable fragment from the last steps in glucoseamine metabolism (Figure 4). Glucoseamine metabolism is required for O-glycosylation and thus its higher abundance substantiates our hypothesis of increased demand for biomolecules in MiaPaCa-2 cells. A likewise pattern could be observed for another amino sugar, N-acetylneuraminic acid. Further, when protein synthesis is elevated, the absolute amount of translation errors would increase. We detected increased levels of aminomalonic [53] acid in MiaPaCa-2 cells and aminomalonic acid might originate from erroneous protein synthesis.

Most importantly, the acquisition of resistance against nab-paclitaxel had a higher impact on the metabolome than acute nab-paclitaxel treatment did. Despite the cell line-specific alteration to the prolonged application during the generation of resistant cell lines, three metabolites were likewise regulated in resistant cells, regardless of the acute treatment. While aspartic acid was reduced in the resistant cell lines, carbamoyl-aspartate was increased (Figure S3A,B). The opposing regulation of these directly connected metabolites suggests an activation of pyrimidine de novo synthesis. The conversion of aspartic acid to carbamoyl-aspartate in pyrimidine synthesis is conducted by the enzyme aspartate transcarbamylase, part of the tri-functional protein carbamoyl-phosphate synthetase 2, aspartate transcarbamylase, and dihydroorotase (CAD) [54].

In summary, there were only small metabolic differences between Panc-1/NPR treated and untreated cells, as well as between MiaPaCa-2/NPR treated and untreated cells supporting that acute nab-paclitaxel does not have a high impact on cellular metabolism. In contrast, resistance against nab-paclitaxel was accompanied by changes in some metabolic pathways. These could be cell line-specific as well as cell line independent.

## 4. Conclusions

Metabolic alterations in cancer cells are either necessary to cover the energetic demand of the cells or they contribute to the aggressiveness of cancer cells [55,56]. Glucose uptake and metabolization through the polyol pathway was linked to a mesenchymal phenotype of the cells [55]. Metabolomics profiling identified an activated polyol pathway in Panc-1 cells, which is in line with high levels of vimentin, confirming a mesenchymal phenotype [47,55]. This pathway was even more active in the nab-paclitaxel resistant cell lines. Since previous studies found that mesenchymal cells are more resistant to paclitaxel treatment and polyol pathway activation goes along with EMT, the upregulation of that pathway possibly contributed to the acquired resistance against nab-paclitaxel. Therefore, a combinatorial therapy of nab-paclitaxel with an aldose-reductase inhibitor might delay the acquisition of nab-paclitaxel resistance in pancreatic cancer [57]. Another phenomenon observed in MiaPaCa2 cells with resistance to nab-Paclitaxel was an increased lactate concentration. In a recent study by Sandforth, Ammar and colleagues, the elevated intracellular accessibility to lactate was linked to chemotherapy resistance in different pancreatic cancer cell lines [58] which could also contribute to the resistance against nab-Paclitaxel.

In general, nab-paclitaxel led to only a few metabolic alterations. This could be explained by the mode of action. Nab-paclitaxel arrests cancer cells in mitosis, whereas other chemotherapeutic agents such as 5-FU and gemcitabine interfere directly with DNA synthesis by acting as an anti-metabolite [28,59]. However, after acquisition of resistance against nab-paclitaxel, metabolic fingerprints differed to the corresponding parental cell lines. A likewise trend observed in both cell lines were a reduction in aspartic acid and an increase in carbamoyl-aspartic acid. This can be explained by an elevated activity of CAD, which is the rate limiting enzyme complex in de novo pyrimidine synthesis. The elevated supply with pyrimidines could favor the acquisition of resistance against chemotherapeutic agents. In line with that is a recent study by Halbrook and colleagues, in which they showed resistance against gemcitabine by extracellular supply of pyrimidines by tumor-associated macrophages [60]. On top of that, de novo pyrimidine synthesis was found to support resistance against several chemotherapeutic agents in triple-negative breast cancer [61]. 

Another aspect that has to be mentioned is the possible existence of sub-populations in cancer cell lines, which could have been selected during the prolonged chemotherapy application [62]. 

In summary, we investigated the metabolic fingerprint of different pancreatic cancer cell lines and showed how metabolism changed after acquisition of chemotherapy resistance. We are the first group evaluating the metabolic adaptions and changes in Panc-1 and MiaPaCa-2 cells being resistant to nab-Paclitaxel using untargeted GC/MS-Analysis. Since it is not clear whether the metabolic alterations are causative, supportive or an epiphenomenon, the putative vulnerabilities of the affected pathways have to be functionally validated in further studies. Such studies might include the establishment of nab-paclitaxel resistant pancreatic cancer cell lines with and without simultaneous inhibition of aspartate transcarbamoylase. Such inhibition can be achieved by the anti-metabolite N-phosphonacetyl-L-aspartic acid [63]. Time-resolved comparison of IC50 values can unveil whether targeting this enzyme could delay acquisition of resistance. If the results of these experiments are successful, patient-derived organoid cultures [64] could be generated and subjected to the same experiment, i.e., establishment of resistance by prolonged nab-paclitaxel treatment with or without aspartate transcarbamoylase inhibition. Such inhibition can also be performed by gene interference. For example, an inducible CAD-knockdown could be established in these cell lines and subjected to resistance acquisition. Afterwards, parental and resistant cell lines could be transplanted into mice, before the following read-outs could be performed: Is there a growth difference between parental and resistant transplanted cells in vivo? Is there a difference in tumor growth upon nab-paclitaxel treatment in transplanted mice? If so, can this nab-paclitaxel resistance be reverted in vivo, when CAD knockdown is induced?

These are important questions to address in further validation studies. However, our results might already aid in better understanding the mechanisms of chemotherapy resistance in pancreatic cancer cells.

## Figures and Tables

**Table 1:** Differences between IC50 values of nab-paclitaxel in parental and resistant MiaPaCa-2 and Panc-1 cells.

Cell Line	Parental IC50	Resistant IC50	Fold Change
MiaPaCa-2	0.0041 nM	0.31 nM	74.7
Panc-1	0.0073 nM	0.25 nM	33.3

## Supplementary Materials

The following are available online at https://www.mdpi.com/2073-4409/9/5/1251/s1, Supplementary Figure S1: Metabolomics analysis of gemcitabine treated MiaPaCa2 and Panc-1 cells. Supplementary Figure S2: Principal component analysis of parental and resistant MiaPaCa-2 and Panc 1 cells. Supplementary Figure S3: Fold change of aspartic acid and N-carbamoyl-aspartic acid. Supplementary data file SD1: GC/MS results of nab-paclitaxel treatment in PDAC cell lines. Supplementary data file SD2: GC/MS results of nab-paclitaxel resistance in PDAC cell lines.
