# Supplementary material for: Metabolic Adaptation during nab-Paclitaxel Resistance in Pancreatic Cancer Cell Lines
Source: Cells. 2020 May 19;9(5):1251. doi: 10.3390/cells9051251 (PMC7290296; doi:10.3390/cells9051251)
Supplement: Supplementary file 1 [file cells-09-01251-s001.zip › Supplementary information.docx]

Article

Metabolic adaptation during nab-paclitaxel resistance in pancreatic cancer cell lines –
Supplementary information

Lukas M. Braun^1,2,#^, Simon Lagies^1,3,4,#^, Jessica Guenzle^2^, Stefan Fichtner-Feigl^2^, Uwe A. Wittel^2,*^, Bernd Kammerer^1,3,5,^*

^1^ Center for Biological Systems Analysis ZBSA, Albert-Ludwigs-University Freiburg, 79104 Freiburg, Germany

^2^ Department of General- and Visceral Surgery, University of Freiburg Medical Center Faculty of Medicine, 79106 Freiburg, Germany

^3^ Spemann Graduate School of Biology and Medicine, Albert-Ludwigs-University Freiburg, 79104 Freiburg, Germany

^4^ Institute of Biology II, Albert-Ludwigs-University Freiburg, 79104 Freiburg, Germany.

^5^ BIOSS Centre for Biological Signalling Studies, University of Freiburg, 79104 Freiburg, Germany

^#^ These authors contributed equally to this paper.

***** Corresponding authors: bernd.kammerer@zbsa.uni-freiburg.de (B.K.); uwe.wittel@uniklinik-freiburg.de (U.A.W.)


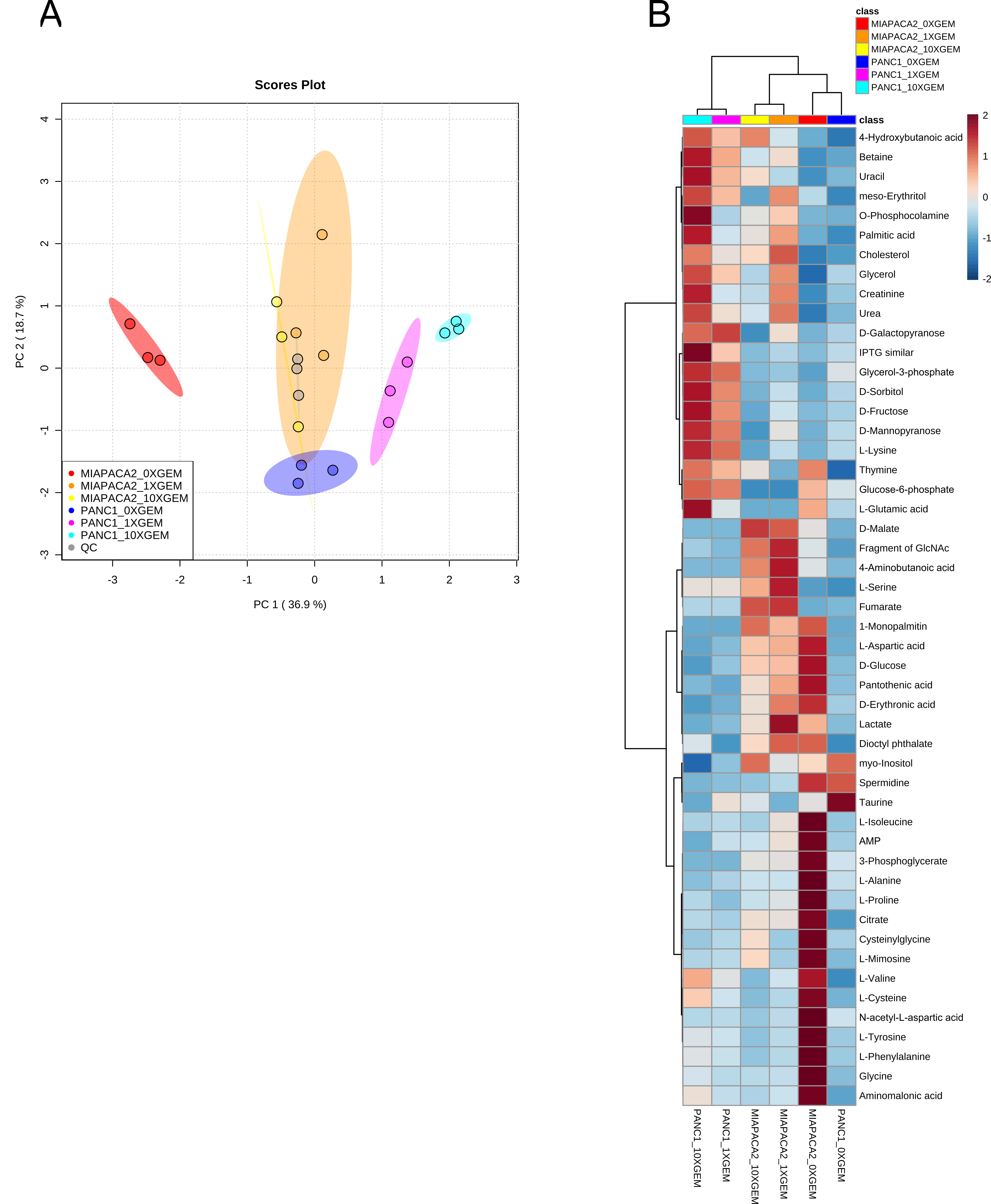


Supplementary figure 1: Metabolomics analysis of gemcitabine treated MiaPaCa2 and Panc-1 cells: **A** principal component analysis of untreated (0xGEM), and treated (1XGEM and 10XGEM) pancreatic cancer cell lines. 1X and 10X Gem indicates treatment for 72 h with one-fold or ten-fold IC50 concentration as assessed by the resazurin viability assay. n=3. B Corresponding heat map analysis of significantly altered metabolites. Range-scaled z-scores are shown. n=3


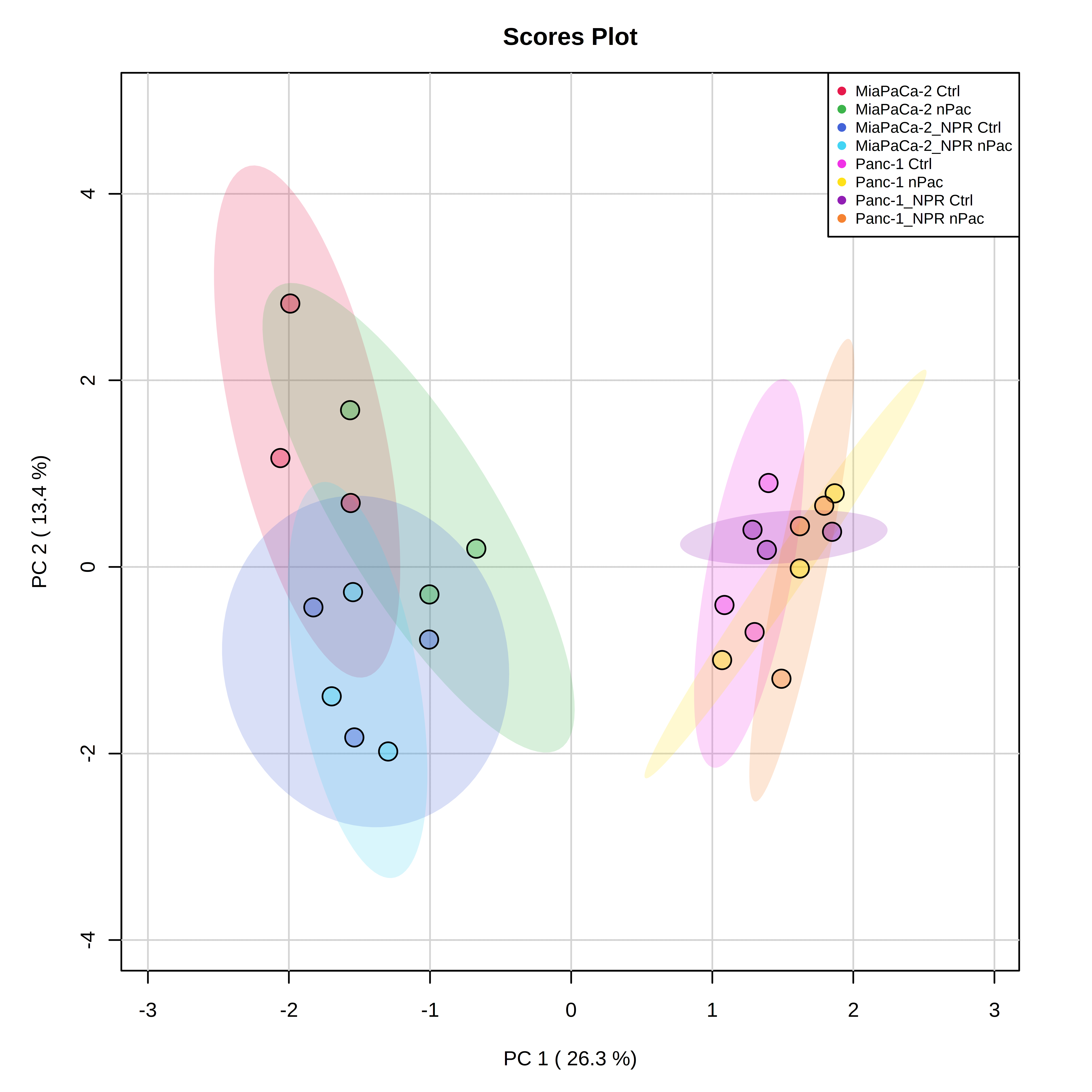


Supplementary Figure 2: Principal component analysis of parental and resistant MiaPaCa-2 and Panc‑1 cells upon chemotherapy treatment for 72 h. nPac: nab-Paclitaxel treated; Ctrl: vehicle treated. n=3

Supplementary Figure 3: Fold change to corresponding ctrl of aspartic acid (A) and N-carbamoyl-aspartic acid (B) in in parental and resistant MiaPaCa-2 and Panc-1 cell lines. Cells were treated (nPac) or not (ctrl) for 72 h. Most abundant ions were normalized for fold change analysis (232.2 *m/z* for A and 257 *m/z* for B, retention times have been proved by analytical standards). Error bars indicate S.E.M. *p-value < 0.05 to untreated parental cell line, ** p-value < 0.01 to untreated parental cell line. n=3
